# AIR QUALITY AND WELL-BEING AMONG MIDDLE-AGED AND OLDER ADULTS IN EAST ASIA

**DOI:** 10.1093/geroni/igz038.991

**Published:** 2019-11-08

**Authors:** Takashi Yamashita, Giyeon Kim, Darren Liu, Anthony R Bardo

**Affiliations:** 1 University of Maryland, Baltimore County, Baltimore, Maryland, United States; 2 Chung-Ang University, Seoul, Korea, Republic of; 3 Des Moines University, Des Moines, Iowa, United States; 4 University of Kentucky, Lexington, Kentucky, United States

## Abstract

There is a well-established link between air quality, an important component of one’s local living environment, and well-being. However, the link between well-being and air quality is largely based on findings from western nations, and evidence from East Asia (where air pollution is a major challenge) is scant. Thus, the present study sheds much needed light on the association between well-being and air quality in four East Asian countries (i.e., China, Japan, South Korea, and Taiwan). Data for this study were drawn from the internationally representative 2010 East Asian Social Survey Health Module. The sample was limited to middle-age and older adults (i.e., 50 years and older) to account for differential exposure across the life course (N = 4,052). Linear regression models with robust standard error estimation and full information maximum likelihood were used to examine associations between four well-being indicators (self-rated health, SF-12 physical health and mental health, and happiness) and self-reported air quality. Results showed that air quality was negatively associated with well-being across East Asian nations --- with self-rated health in Japan (b = -0.09, p < 0.05) and Taiwan (b = -0.14, p < 0.05); physical health in Japan (b = -0.96, p < 0.05); mental health in China (b = -1.05, p < 0.05) and Japan (b = -1.49 , p < 0.05); and happiness in China (b = -0.07, p < 0.05). Possible explanations underlying these distinct national patterns and strategies to enhance well-being through environmental and behavioral interventions are discussed.

